# Relative expression of hormone receptors by endothelial and smooth muscle cells in proliferative and non-proliferative areas of congenital arteriovenous malformations

**DOI:** 10.1186/s40001-023-01436-5

**Published:** 2023-10-20

**Authors:** A. M. Utami, J. B. G. Halfwerk, O. J. de Boer, C. Mackaaij, D. R. Pabittei, C. M. A. M. van der Horst, L. B. Meijer-Jorna, A. C. van der Wal

**Affiliations:** 1 Department of Pathology, Amsterdam University Medical Center-Location AMC, University of Amsterdam, Amsterdam, The Netherlands; 2 Department of Anatomical Pathology, Faculty of Medicine, Hasanuddin University, Makassar, Indonesia; 3 Department of Plastic Surgery, Amsterdam University Medical Center-Location AMC, University of Amsterdam, Amsterdam, The Netherlands; 4 Department of Physiology, Faculty of Medicine, Hasanuddin University, Makassar, Indonesia; 5 Symbiant Pathology Expert Center, NWZ- Noordwest Ziekenhuisgroep, Alkmaar, The Netherlands; 6 Department of Anatomy, University Medical Center Utrecht, Utrecht, The Netherlands

**Keywords:** Arteriovenous malformations, Hormone receptor, Microvascular proliferation, Vascular malformations

## Abstract

**Background:**

Episodic growth due to microvascular proliferations (MVP) has been reported in congenital arteriovenous malformations (AVM), which are normally quiescent lesions composed of mature malformed vessels. Since AVM also may worsen under conditions of hormonal dysregulation, we hypothesized that hormonal influences may stimulate this process of vasoproliferative growth through potential interactions with hormone receptors (HR).

**Methods:**

13 Cases of AVM tissue with histologically documented vasoproliferative growth were analyzed quantitatively for the presence and tissue localization of estrogen receptor (ER), progesterone receptor (PGR), growth hormone receptor (GHR) and follicle-stimulating hormone receptor (FSHR) in relation to resident cells of interest (endothelial cells (EC), smooth muscle cells (SMC) and mast cells (MC)) by applying multiplex immunohistochemistry (IHC) staining. Expression patterns in lesions with MVP and mature vessels were quantified and compared. Available fresh frozen tissues of 3 AVM samples were used to confirm the presence of HR using Reverse-Transcriptase quantitative Polymerase Chain Reaction (RT-qPCR).

**Results:**

All four HR studied were expressed in all cases within EC and SMC in areas of MVP and mature vessels, but not in normal skin tissue. ER, GHR, and FSHR showed more expression in EC of MVP and in SMC of mature vessels. RT-qPCR confirmed presence of all 4 HR in both areas.

**Conclusion:**

Expression of ER, PGR, GHR, and FSHR in vasoproliferative areas of congenital AVM could explain onset of sudden symptomatic growth, as has observed in a subpopulation of patients. These findings may have implications for eventual anti-hormonal targeted therapy in the lesions involved.

**Supplementary Information:**

The online version contains supplementary material available at 10.1186/s40001-023-01436-5.

## Introduction

Arteriovenous malformations (AVM) of skin and soft tissues are rare congenital vascular lesions, arising from dysmorphogenesis during embryogenesis. They usually have a slowly progressive growth commensurate with the growth of the patient, and do not regress overtime [1–3]. Compared to other types of simple vascular malformations, AVM are the most problematic lesions in terms of clinical behaviour due to their high flow characteristics [2]. Despite an overall quiescent type of growth, AVM can enlarge disproportionally over time and may become symptomatic due to trauma, inflammation, thrombosis, hormonal changes, or complications related to pressure on surrounding tissues [4, 5].

Episodic growth of AVM has also been attributed to occurrence of areas of microvascular proliferations (MVP) [4–6]. Meijer-Jorna et al. have reported presence of MVP areas in 30% of 107 resection and amputation specimens of AVM obtained from patients who were treated for symptomatic disease [4, 5]. In 2009, Duyka et al. reported that a disproportional growth of the AVM mass occurs during episodes of hormonal disbalance such as adolescence, puberty, pregnancy or the use of hormonal contraception [7–11]. Such observations raise the question whether hormonal stimulation or dysregulation in patients with AVM could be involved in stimulating or aggravating a process of angiogenesis. Indeed, the highest risk of progression to a higher Schobinger Stage occurs during adolescence, suggesting that circulating hormones might contribute to AVM proliferation [12, 13].

Several studies have demonstrated that specific hormone receptors (HR) such as progesterone receptor (PGR), growth hormone receptor (GHR), and follicle-stimulating hormone receptor (FSHR) were present in vascular tumors and vascular malformations [5, 7, 14]. In these studies, expression of GHR, FSHR, and PGR was reported mostly in endothelium of vascular malformations. GHR was found in the endothelial/perivascular area of vascular malformations, and also in the stroma of control tissues [5]. FSHR was found mostly in endothelial cells of vascular anomalies, but not in normal control specimens [14]. PGR was found in lesional endothelial cells (EC) and smooth muscle cells (SMC) [7]. Estrogen receptors (ER) could not be detected in the vessel walls of vascular malformations [5, 7], but interestingly were reported to be present in infantile hemangiomas (IH), which are characterized by an initial stage of growth due to microvascular (capillary) proliferations [15].

Hormones such as estrogen and follicle-stimulating hormone (FSH) have been described to be involved in angiogenesis. Several studies have reported a positive correlation between ER expression, angiogenic activity, and an invasive growth pattern of breast tumors [16–18]. These findings support the angiogenic effect of estrogen mediated by ER. Moreover, a study reported that FSHR was found in pericytes of IH, but it remains unclear whether this concerns the proliferation phase of the these lesions [19]. Other studies also found that FSHR expressed on the EC of the blood vessels of tumors like lung, breast, colon, kidney, and metastatic prostate carcinoma [20, 21].

Expression of several HR on the vascular walls of several types of vascular malformations including AVM has been described only in mature vessels of vascular malformations, but was not investigated specifically in areas of MVP [5, 22]. Hypothetically, an increase in expression of HR in vasoproliferative areas could explain the expansive growth of AVM types of vascular malformations during episodes of hormonal excess.

Therefore, the present study was designed to immunohistochemically investigate the expression of several types of HR in which a vasoproliferative response had occurred. Archived paraffin blocks of large AVM resection specimens were selected which contained histologically proven vasoproliferative areas amidst the vessels of the pre-existent arteries and veins of the malformation. Immunostaining of ER, PGR, GHR and FSHR was applied, followed by comparing the relative expression of the respective HR in both areas of interest (MVP versus pre-existent mature vessel areas). Since several cell types can be involved in angiogenesis, particularly EC, SMC, and mast cells (MC), we also investigated the co-localization of HR expression in these cell types. Potentially this could improve our knowledge on angiogenic growth in AVM lesion, in addition to the previously known insight of angiogenic factors released by mast cells [23–25].

## Methods

### Study materials

Paraffin blocks were retrieved from the archives of the Department of Pathology at Amsterdam University Medical Center (Amsterdam UMC), location AMC, a referral center for management of vascular malformations. The study materials were selected from a file of anonymized 37 AVM cases with histologically proven present proliferative areas amidst the conglomerates of mature vessels. Proliferative areas where defined as solid areas of densely packed capillary vessels lined with plump endothelium. Proliferative activity was confirmed by increased Ki67 labelling index of the microvessels [4, 26]. On the basis of availability and quality of the tissue, 13 samples could be included in the study. The average age of patients was 25.07 ± 13.9, and 69.2% (*n* = 9) were females (Table 1). Additionally, five paraffin blocks of normal skin and subcutis specimens were subjected to the same procedures of immunostaining for comparison of HR expression patterns. Positive control tissues for immunohistochemistry (IHC) consisted of paraffin blocks containing breast carcinoma, kidney and testis. Finally, fresh frozen tissues (FFT) of 3 AVM samples, stored at – 80 ℃, were also available and used for reverse transcriptase-quantitative polymerase chain reaction (RT-qPCR) analysis of HR.

**Table 1:** Patient characteristics of AVM samples with MVP used for IHC (*n* = 13) and for RT-qPCR (*n* = 3)

IHC	MEAN ± SD	
Age of patients (years)	25.07 ± 13.9	
	N	%
Sex		
Females	9	69.2
Males	4	30.8
Topographic location		
Arm	1	7.6
Ear	2	15.3
Eye	1	7.6
Face	1	7.6
Foot	1	7.6
Leg	1	7.6
Lip	3	23
Nose	2	15.3
Skin	1	7.6

RT-qPCR	MEAN ± SD	
Age of patients (years)	30 ± 8	
	N	%
Gender		
Females	1	33.3
Males	2	66.7

Serially cut sections (4 µm) were stained with Hematoxylin & Eosin (H&E) and Elastic van Gieson (EvG) stains, respectively, to confirm the diagnosis of AVM and presence of representative areas of MVP. Adjacent sections were mounted for IHC staining.

#### Immunohistochemistry

The following antibodies were used: monoclonal rabbit anti-Ki67 (proliferating cells, ThermoFisher Scientific, Fremont, CA, USA), mouse anti-estrogen receptor (targeted ER-α, DAKO, Glostrup Denmark), monoclonal mouse anti-progesterone receptor (DAKO), polyclonal rabbit anti-growth hormone receptor (ProSci, Poway, CA, USA) and monoclonal mouse anti-follicle stimulating hormone receptor (NSJ Bioreagents, San Diego, CA, USA), mouse monoclonal anti-CD31 (endothelial cells, DAKO), mouse monoclonal anti-smooth muscle actin/SMA-1 (smooth muscle cells, DAKO) and mouse monoclonal anti-Tryptase (mast cells, DAKO).

#### Immunohistochemistry single staining

Ki67 was applied using single IHC staining to detect vasoproliferative areas. In short, sections were dewaxed in xylene and rehydrated in graded-alcohols prior to antigen retrieval with heat-induced epitope retrieval (HIER) in a PT Module (ThermoFisher Scientific, Waltham, MA, USA) using Tris–EDTA buffer (ThermoFisher Scientific). Endogenous HRP was blocked in methanol + H_2_O_2_ (30% diluted in methanol to a 0.3% solution). Incubation with primary antibody (Ki67), followed by horseradish peroxidase (HRP) anti-rabbit polymer secondary antibody (Brightvision, Immunologic, Duiven, The Netherlands). HRP enzyme activity was detected in brown using DAB plus (Brightvision, Immunologic), and counterstained with Hematoxylin.

#### Immunohistochemistry quadruple multiplex staining

IHC quadruple multiplex staining was performed to investigate the distribution of the HR (ER, PGR, GHR, FSHR) by resident cells (EC, SMC, and MC). Essentially, the staining was performed as described above in single IHC staining, with the only difference that the Vector Novared (Vector Labs, Burlingame, CA, USA) was used as a chromogen. Sections were counterstained with Hematoxylin, dried on a hotplate, coverslip sealed with Glycerol/Gelatine (Sigma–Aldrich, St.Louis, Missouri, USA) and digitized using a Philips IntelliSite ultra-fast Scanner (Philips Digital Pathology Solutions, Best, The Netherlands). After scanning, a second staining round was performed by removing the coverslip in hot water and subjecting the sections to a stripping buffer (Tris-SDS + beta mecapthoenatol) in which the colours and immune complexes were eluted from the sections. After washing, the sections were stained with a different antibody and digitized again as described above [27]. The process of staining and de-staining was repeated according to a predetermined procedure, optimal sequence: the HR was always performed as first staining, followed by CD31, SMA-1, and Tryptase. Off note, positive and negative control staining confirmed the specificity of the antibodies and used techniques.

### Analysis of hormone receptor co-localization

In each quadruple multiplex immunostained section containing immunostained EC, SMC, MC and one of the respective HR, regions of interest (ROI) consisting of MVP areas and of mature vessels were selected measuring ± 1.56 mm^2^. Colocalization of the HR antibody with resident cell specific antibodies (EC, SMC, and MC) was quantified by calculating the Manders Overlap Coefficient (MOC), a quantitative co-localization analysis method [10, 11]. In each ROI, the amount of immunopositive pixels for the HR, the resident cell type, and the amount of overlapping (thus double stained) pixels were counted. MOC M1 calculates the ratio of HR-double stained pixels and the total amount of HR pixels in the ROI, and is a measure of the distribution of the HR over the resident cell types. MOC M2 on the other hand, calculates the ratio of HR positive resident cell stained pixels and the total tissue area of resident cells, thus representing a measure of the amount of HR expressed by each resident cell type [28]. Digital image analysis was performed using Image Pro Premier (vs 9.3, Media Cybernatics, Rockville, MD, USA).

### Statistical analysis

To compare HR expression in different tissue areas of the malformations and its colocalization in specific cell types, a normality test was performed with Shapiro–Wilk test, and a non-parametric Mann–Whitney test were used. Statistical analysis was performed with SPSS 26.00 (IBM Corporation, Armonk, NY, USA).

### Reverse transcriptase – quantitative polymerase chain reaction

RNA isolation was applied on each available frozen AVM samples and control tissues. 30 sections of 20 μm were cut, and RNA was isolated using RNABee (Bio-Connect, Huissen, the Netherlands) and the RNeasy Mini kit (Qiagen, Hilden, Germany) according to manufacturer’s instructions. Then, RNA concentration was determined at 260/280 nm using a Nanodrop spectrophotometer (Thermo Fisher Scientific) to create cDNA [29].

Single-stranded cDNA was synthesized from 500 ng total RNA at a temperature of 40 ℃ for one hour using random hexamer primers (Promega, Madison, WI) and M-MLV Reverse Transcriptase (Invitrogen, Waltham, MA). Finally, four pairs primers (Table 2) were developed for the HR (forward and reverse) and applied to cDNA. RT-qPCR was preformed using the CFX Touch Real-Time PCR detection system. LinRegPCR method was used for quantification method [30, 31] Glyceraldehyde 3-phosphate dehydrogenase (GAPDH) and Ribosomal Protein L13a (RPL13A) were used as reference genes. SensiFAST^™^ SYBR^®^ No-ROX Kit (Meridian Bioscience, Memphis, USA) was used in a 10 μl reaction. A non-quantitative RT-PCR for HR was applied on breast cancer, kidney, and testis tissue, which were all positive, while a sample of normal skin tissue was negative.

**Table 2:** Primer sequences used for RT-qPCR

PCR primers	Sequence(s)	Annealing temperature
ESR1-forward	TGGGAATGATGAAAGGTGGGAT	53 ℃
ESR1-reverse	GGTTGGCAGCTCTCATGTCT	
PGR-forward	CAGCCAGAGCCCACAATACA	55 ℃
PGR-reverse	GTTGTGCTGCCCTTCCATTG	
GHR-forward	GCGCAGACGCGAACC	51 ℃
GHR-reverse	AGGCTCCTTAGAAGAATTTGTCTTT	
FSHR-forward	ATAAGGGCACTGTGTGGAGC	53 ℃
FSHR-reserve	TGGTGAGGACAAACCTCAGTT	

## Results

### Histology

**Fig.1:**
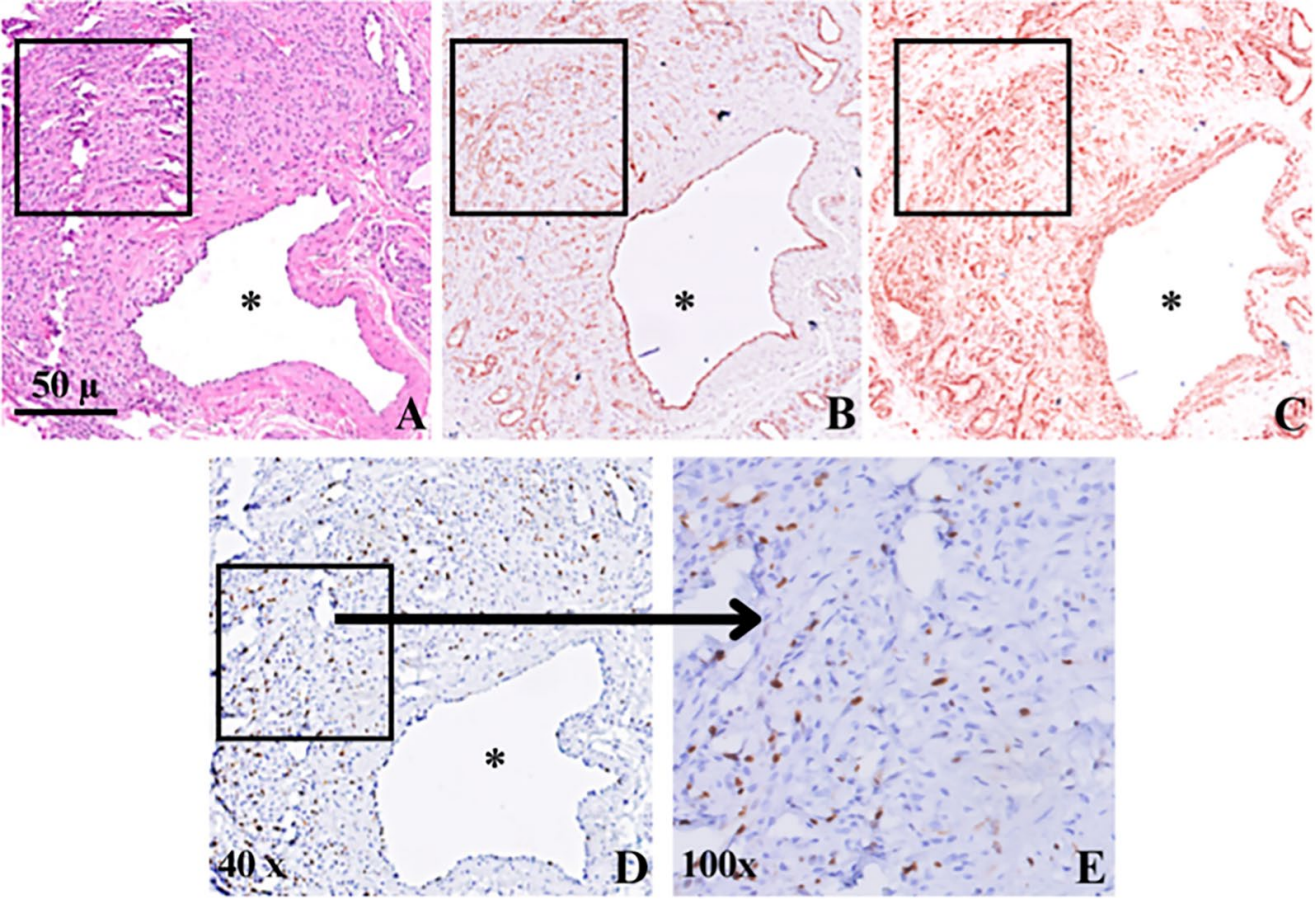
Serial sections of a case of AVM lesion stained with (**A**) H&E and IHC with (**B**) CD31 **(C)** SMA-1 (**D**, **E**) Ki67 antibody. Black squares in figs **A**,** B**, **C**, **D** indicate area with MVP, which is enlarged in fig (**E**), showing many Ki67 immunopositive cells in the vessel walls of microvessels immunopositive nuclear staining. Asterisk (*) is in the lumen of a large blood vessel of the AVM

Histopathology using HE and Elastic van Gieson stains showed in all 13 cases the presence of both mature, but sometimes malformed arteries with a tortuous course (several cross sections through arteries) in combination with conglomerates of malformed veins. Presence of intimal thickening and dilatation of veins was also observed regularly suggestive for aberrant (high) flow characteristics of the lesions. These findings were consistent with the diagnosis AVM. In addition, all 13 samples showed areas of closely packed capillary vessels with plump endothelium and MVP and high labeling index of Ki67 (more than 5 positive cells/mm^2^) amidst the mature vessels of the malformations [4, 26] (see Fig. 1).

### Hormone receptor expression

**Fig.2:**
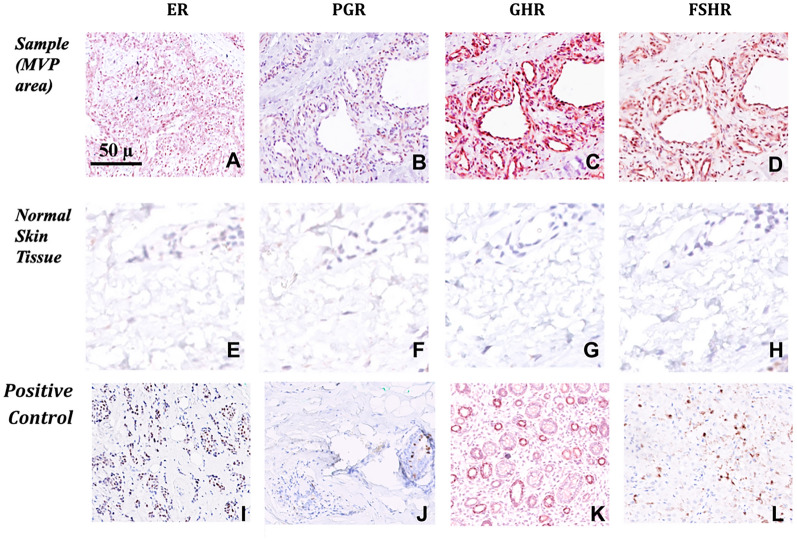
AVM sample immunostained with antibodies reactive with ER (**A**), PGR (**B**), GHR (**C**), FSHR (**D**), compared with reactivity of the same antibodies in normal human skin tissue (**E**–**H**). Note the abundant expression of HR in MVP the area, but not in normal skin. **I**-**J**: Immunohistochemical staining of ER, PGR, GHR, and FSHR, respectively, on positive control tissues: breast cancer (**I**), breast cancer (**J**), kidney (**K**), testis (**L**)

All 4 types of HR (ER, PGR, GHR and FSHR) were found to be expressed in MVP areas of all 13 cases, albeit in variable amounts. Specifically, PGR and GHR showed a nuclear type of positive immunostaining, while ER and FSHR showed a nuclear and in addition cytoplasmic pattern of immunostaining in all lesions. In normal skin tissue, no expression with any of the HR was observed (for representative examples see Fig. 2).

**Fig.3:**
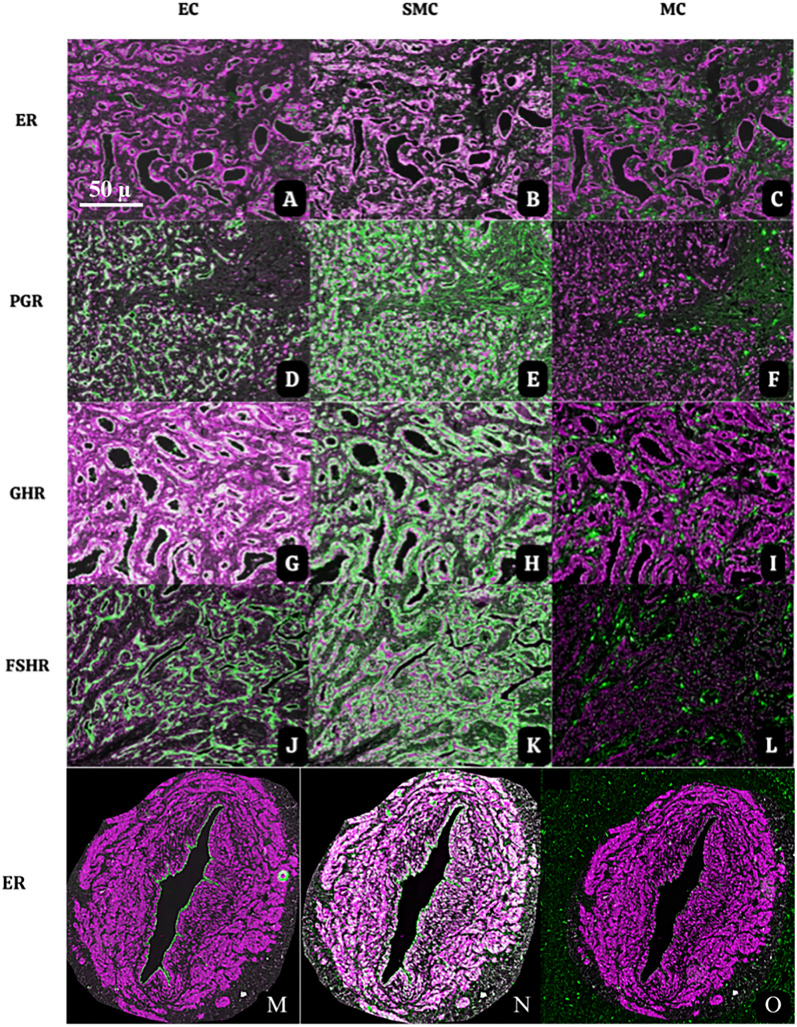
Examples of multiplex immunohistochemistry staining of MVP areas in AVM showing colocalization of ER (**A**, **B**, **C**), PGR (**D**, **E**, **F**), GHR (**G**, **H**, **I)**, and FSHR (**J**, **K**, **L**) (all in magenta) in resident cells EC, SMC, (all in green) in MVP area. **MNO**: Multiplex immunostaining showing ER expression (magenta) co-localized with EC (**M**), SMC (**N**) MC (**O**) (all in green) on a large mature blood vessel of AVM. Of note: Colocalization of immunostains in EC and SMC appears as white colour in all the figures. There are no colocalizations visible in MC (**C**, **F**, **I**, **L**, **O**)

To investigate the cellular source of the HR in the tissue specimens, IHC quadruple multiplex staining was performed, showing staining of HR in combination with cell specific markers (EC, SMC, and MC, respectively). All 4 types of HR were expressed on EC and SMC, and none on MC. Overall, most abundant expression was observed in SMC (for representative examples see Fig. 3).

**Fig.4:**
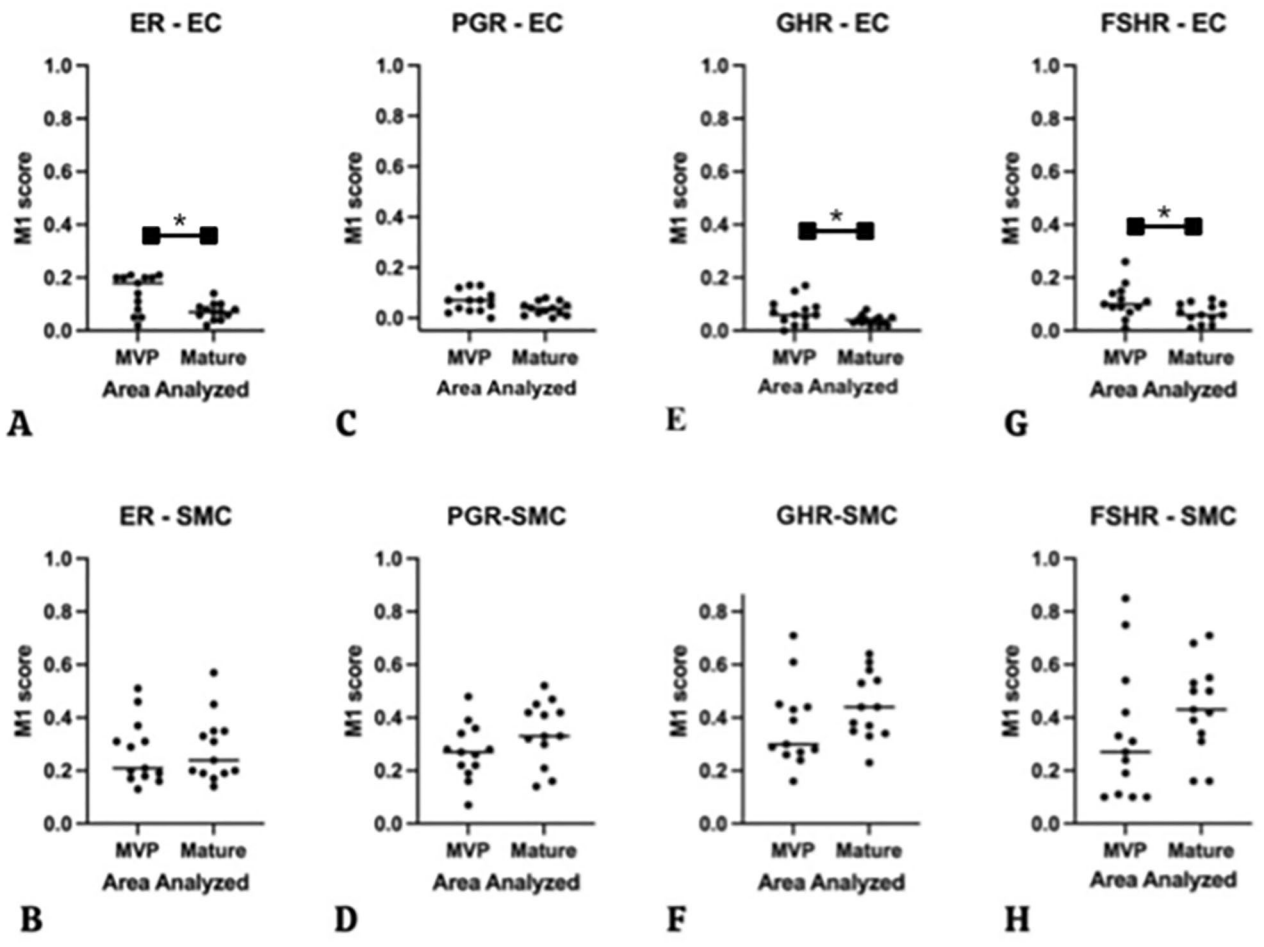
8 Dot plot graphs showing M1 scores of immunostaining of HR in EC and SMC of all 13 cases of AVM comparing MVP areas versus mature vessels areas of the lesions ER in EC & SMC (**A**, **B**), PGR in EC & SMC (**C**,** D**), GHR in EC & SMC (**E**, **F**), and FSHR in EC & SMC (**G**, **H**). Statistically significant results were found in ER-EC (**A**), GHR-EC (**E**), and FSHR-EC (**G**). *: *P* < 0.05 (Mann Whitney *U* test)

To compare the relative amounts of HR expression of resident cells in areas of proliferative with adjacent non-proliferative AVMs, MOC M1 and M2 scores were determined (see Additional file 1: Table S1, S2, S3, S4) M1 scores of ER, GHR, and FSHR in EC were significantly higher in MVP areas when compared to non-proliferative areas (Fig. 4), which reflects the presence of significantly increased numbers of the respective HR+ EC in MVP areas. Only PGR showed no significant differences between the two areas. M1 scores of all HR were higher in SMC than in EC, there were no significant differences noted between proliferative and mature areas.

**Fig.5:**
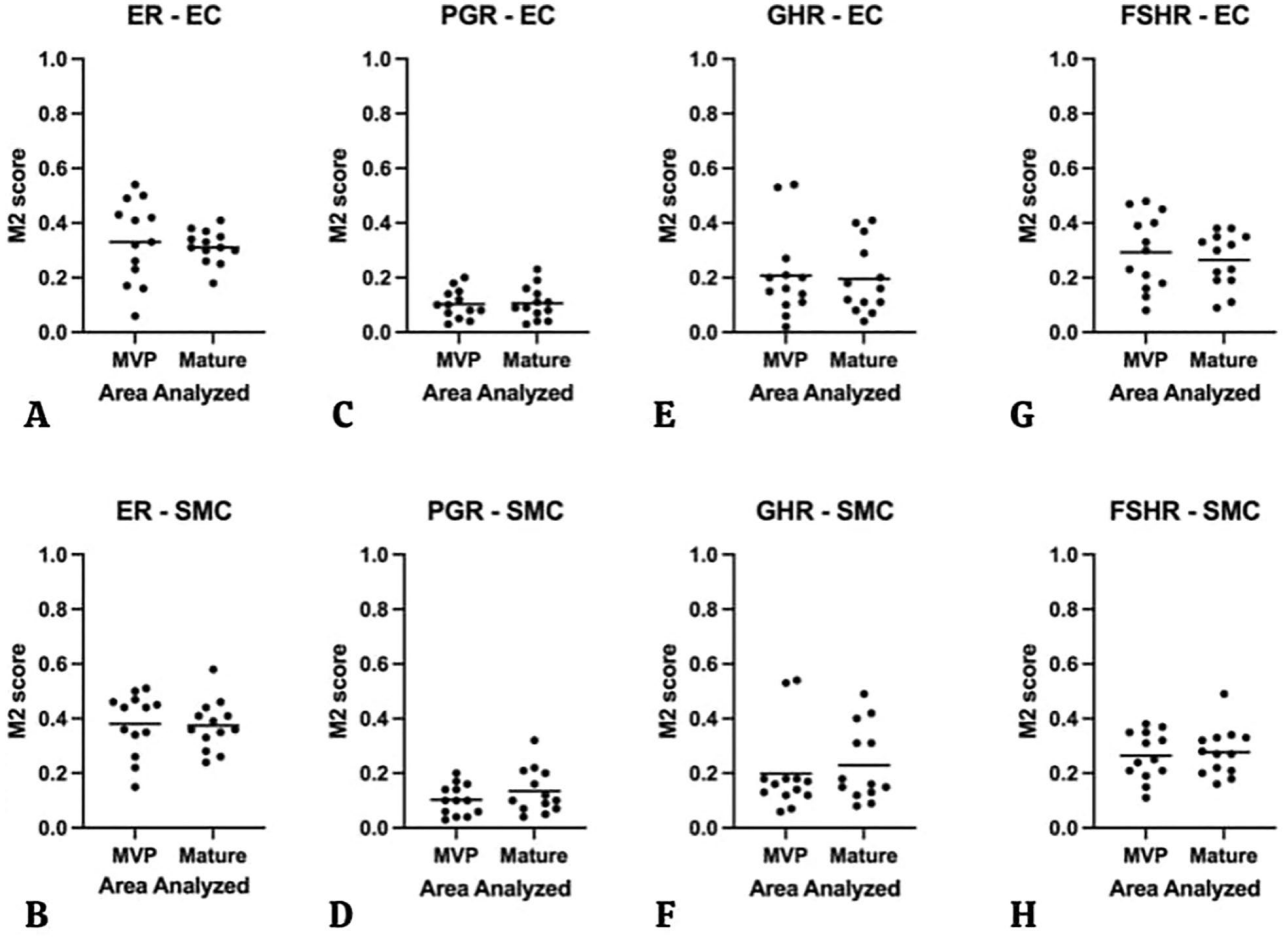
8 Dot plot graphs showing M2 scores comparing MVP areas with mature vessels areas of each AVM lesions of all 4 types of HR in each type of resident cells. ER in EC & SMC (**A**, **B**), PGR in EC & SMC (**C**, **D**), GHR in EC & SMC (**E**, **F**), and FSHR in EC & SMC **(G**, **H).** There is no significant result of M2 score comparison of MVP areas with mature vessels in EC and SMC. There we no significant differences in M2 score observed between MVP and mature areas (Man Whitney *u*)

On the other hand, the corresponding M2 scores did not show any significant differences between proliferative and non-proliferative areas (Fig. 5), indicating that the levels of HR expression by the resident cells were similar in proliferative and mature areas.

### RT-qPCR for hormone receptor

**Fig.6:**
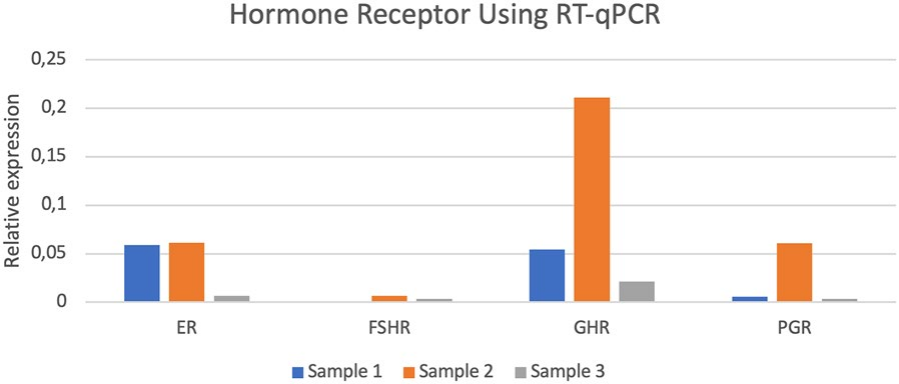
Bar graph showing relative expression (vertical axis) of all 4 hormone receptors (horizontal axis) using Reverse Transcription quantitative Polymerase Chain Reaction

Finally, RT-qPCR was performed to acquire additional evidence of the synthesis of ER, PGR, GHR, and FSHR in AVM lesions containing proliferative areas in the available 3 FFT samples. This study confirmed positive expression of ER, PGR, GHR, and FSHR in all samples quantitatively with results between 0 and 0.22 (Fig. 6), except for one sample which scored negative for FSHR.

## Discussion

Expansive growth related to onset of MVP has been reported in a substantial fraction of arteriovenous malformations (AVM) [6]. The present study reports the expression pattern of several types of HR in relation to vasoproliferative activity. We found that ER, PGR, GHR, and FSHR expression could be detected immunohistochemically in all lesions, both in histological areas of closely packed immature microvessels representing proliferative growth, and in the thick-walled arteries and veins of the malformations. This was further confirmed by RT-qPCR analysis in 3 lesions of which frozen tissue was available. On the other hand, expression of HR appeared to be absent in vessels of samples of normal skin tissue, which indicates that HR expression could be a specific feature of the malformations, albeit not solely for the proliferative component.

HR expression has been reported previously in tissue specimens of vascular malformations, especially in AVM [5, 7, 14, 32]. Kulungowski et al. reported that GHR was primarily present in the endothelium/perivasculature of malformations [5], Maclellan et al. reported that FSHR was found in the endothelium [14], and Duyka et al. reported that AVM, VM, and LM specimens showed positive staining for PGR within the nuclei of EC and SMC of the malformed vessels [7]. However, occurrence of MVP areas was not noticed or specifically described in these reports, so relative expression of HR in proliferative areas has not been published thus far.

Estrogens are considered to be the most-involved hormones in puberty of both male and female [7]. Kulungowski et al. [5] reported the expression of ER in a series of malformations, which included also a case of AVM, but expression was less abundant compared to GHR expression in their samples. Duyka et al. [7] and Ventejou et al. [22] reported negative expression for ER in vascular malformations. In our series, ER expression was found in all types of vessels involved, including arteries, veins and microvessels, albeit in variable proportions. In general, estrogen may promote angiogenesis in vivo and in vitro through several mechanisms, including activation of ER and consequently enhance the pathophysiological process of angiogenesis in EC [5, 33, 34]. Estrogen also stimulates vascular endothelial growth factor, which is involved in neovascularization regulating angiogenesis [24, 35]. Additionally, our finding of concomitant expression of ER and GHR in lesions could imply that estrogen affects GHR functionally [5, 24, 36]. Estrogen stimulates growth hormone (GH) and its receptor in SMC, and proliferation and migration of EC [37, 38]. We found indeed ER expression both in EC and in SMC (Fig. 4A, B). Generally, the staining pattern of ER was mostly nuclear, although we also found additional cytoplasmic staining. This cytoplasmic pattern meets the requirements of ‘positive assessment of cytoplasmic ER’ as described in a previous publication [39].

GH also tends to have a role during puberty, when levels of GH increase rapidly [22]. Increased levels of GH are associated with altered circulating levels of angiogenic factor affecting endothelial cell function [40]. Kulungowski et al. showed an elevated expression of GHR, but no expression of PGR in vascular malformations compared to control samples [5]. Their finding of GHR expression is in line with our findings in the present study, supporting the view that GH may have a role in the expansion of vascular malformations.

Hormonal influence through progesterone in AVM lesions was supported by Duyka et al. who found that 83% of AVM samples in their study group showed diffusely positive PGR expression in EC and SMC compared to controls [7]. However, a possible contribution of progesterone to vasoproliferations in AVM is still unclear, as progesterone is a less potent endothelial mitogen during neovascularization compared to estrogen [3]. Indeed, in our series, PGR showed no significant differential expression between proliferative and non-proliferative areas.

Maclellan et al. have reported the expression of FSHR in a diverse series of vascular anomalies, which included different types of vascular malformations including AVM, and also benign vascular tumors, whereas vascular control tissues were negative. Among these, the highest levels of expression were found in the proliferative stages of IH, which is characterized by abundant microvascular proliferations [14]. The authors argued that the secretion pattern of the hormone, FSH, correlates with the growth cycle in IH, increasing after birth when IH proliferates rapidly and decreasing later when the growth of IH slows. And of note, FSH surges again during adolescence, when AVM are most prone to worsen clinically [14]. Our observation of FSHR expression in EC of MVP areas, might support the view that FSHR plays an important role in AVM expansion.

Taken together, previous publications and this study have provided sample evidence for expression of hormone receptors by vascular wall cells of AVM lesions. At the same time, vascular malformations are likely to grow under hormonal influences as has been noticed clinically during adolescence and pregnancy [7]. Moreover, growth of vascular lesions under hormonal stimulation can be explained by the absence or scarcity of these receptors in normal vascular tissues as shown in this study and others [5, 7, 14, 19]. HR receptors are not only expressed on vascular wall cells of mature AVM vessels but also on vascular wall cells of proliferating microvessels, resulting in high tissue densities of especially positive EC in MVP areas (Fig. 3), which is a major finding of this study, and we postulate that they could provide a target area for rapid expansive growth under hormonal influence. Although we only focused on ER, PGR, GHR, and FSHR expression, other studies have reported on androgen receptor expression [5, 22].

It is assumed that the vasoproliferative response represents a reactive phenomenon, which can be induced by external factors such as inflammation, trauma, and especially circumstances of tissue hypoxia as has been published previously [41–48]. The high EC: SMC ratios that we found in proliferative areas opposed to the mature areas of malformations suggests that EC are the most important target for hormonal influences, stimulating the process of angiogenesis. We investigated also the expression in interstitial MC, which occur in high numbers in proliferative IH and in MVP areas of AVM, and are considered to exert important angiogenic effects through production of various angiogenic peptides [4, 49]. We found hardly no expression of HR in MC (see Fig. 3). Expression of ER and PGR has been reported previously in MC by other studies [50, 51], but not specifically in lesions of AVM. Moreover, Hou et al. showed ER expression in MC of IH samples, but they reported that these MC might be involved in the regressive stage, and not in proliferating stage of IH [15]. This needs further research as literatures on this subject of HR in MC is very limited.

## Limitation

The number of samples included in this study is limited since the study required a highly specific case population. AVM are rare congenital vascular malformations, which require surgical resection only in case of serious symptoms. Moreover, the occurrence of histologically proven MVP occurs only in a subpopulation of the cases, which resulted in a total of only 13 cases.

## Conclusion

The present study advanced our knowledge on angiogenesis and hormonal involvement in the progression of AVM, since ER, PGR, GHR, and FSHR were expressed not only in the mature malformed vessels of AVM but also in the vasoproliferative areas of these lesions. Based on these findings, we propose that the growth of vascular malformations in situations of hormonal dysbalance could be explained by the expression of hormone receptors on vessels of the malformations, in which MVP components may participate. These MVP areas, characterized by high vascular densities, may serve as a target area for episodic rapid growth under such hormonal dysbalanced situations. Unfortunately, due to the anonymized status, no information was available on the hormonal status of the patients involved in this study, but these findings could be useful for ongoing research on a molecular level to determine the definitive association between hormones and angiogenesis in AVM, and development of targeted therapies for recurrence AVM by reducing the use of hormonal therapy and preventing the use of hormonal drugs or contraception.

### Supplementary Information

**Additional file 1: Table S1**. M1 Score for 4 HR in EC between MVP and mature vessels area.** Table S2.** M1 Score for 4 HR in SMC between MVP and mature vessels area.** Table S3.** M2 Score for 4 HR in EC between MVP and mature vessels area.** Table S4.** M2 Score for 4 HR in SMC between MVP and mature vessels area.

## Data Availability

The data used to support the findings of this study are included within the article.

## Abbreviations

AVM: Arteriovenous malformations
EC: Endothelial cells
ER: Estrogen receptor
EvG: Elastic van Gieson
FFT: Fresh frozen tissues
FSH: Follicle-stimulating hormone
FSHR: Follicle-stimulating hormone receptor
GAPDH: Glyceraldehyde 3-Phosphate Dehydrogenase
GH: Growth hormone
GHR: Growth hormone receptor
H&E: Hematoxylin & Eosin
HIER: Heat-Induced Epitope Retrieval
HR: Hormone receptors
HRP: Horseradish Peroxidase
IH: Infantile hemangioma
IHC: Immunohistochemistry
MC: Mast cells
MOC: Manders Overlap Coefficient
MVP: Microvascular proliferations
PGR: Progesterone receptor
ROI: Regions of Interest
RPL13A: Ribosomal Protein L13a
RT-qPCR: Reverse-Transcriptase quantitative Polymerase Chain Reaction
SMC: Smooth muscle cells
