# Supplementary material for: Relative expression of hormone receptors by endothelial and smooth muscle cells in proliferative and non-proliferative areas of congenital arteriovenous malformations
Source: Eur J Med Res. 2023 Oct 20;28:449. doi: 10.1186/s40001-023-01436-5 (PMC10588228; doi:10.1186/s40001-023-01436-5)
Supplement: Supplementary file 1 — Additional file 1: Table S1. M1 Score for 4 HR in EC between MVP and mature vessels area. Table S2. M1 Score for 4 HR in SMC between MVP and mature vessels area. Table S3. M2 Score for 4 HR in EC between MVP and mature vessels area. Table S4. M2 Score for 4 HR in SMC between MVP and mature vessels area. [file 40001_2023_1436_MOESM1_ESM.docx]

| **No** | **Age** | **Sex** | **Topography** | **Hormone Receptor** | | | | | | | |
| --- | --- | --- | --- | --- | --- | --- | --- | --- | --- | --- | --- |
|  |  |  |  | **ER** | | **PGR** | | **GHR** | | **FSHR** | |
|  |  |  |  | **MVP** | **Mature** | **MVP** | **Mature** | **MVP** | **Mature** | **MVP** | **Mature** |
| 1 | 2 | F | Arm | 0,21 | 0,08 | 0,13 | 0,07 | 0,17 | 0,06 | 0,12 | 0,07 |
| 2 | 18 | F | Nose | 0,2 | 0,08 | 0,07 | 0,05 | 0,07 | 0,05 | 0,15 | 0,09 |
| 3 | 16 | F | Foot | 0,21 | 0,07 | 0,09 | 0,04 | 0,06 | 0,02 | 0,09 | 0,02 |
| 4 | 48 | F | Face | 0,2 | 0,07 | 0,13 | 0,02 | 0,10 | 0,04 | 0,14 | 0,02 |
| 5 | 18 | M | Skin | 0,18 | 0,1 | 0,12 | 0,05 | 0,05 | 0,03 | 0,26 | 0,1 |
| 6 | 42 | F | Lip | 0,11 | 0,09 | 0,07 | 0,08 | 0,15 | 0,03 | 0,04 | 0,05 |
| 7 | 30 | M | Leg | 0,05 | 0,04 | 0,07 | 0,03 | 0,08 | 0,04 | 0,07 | 0,06 |
| 8 | 27 | F | Lip | 0,2 | 0,06 | 0,02 | 0,02 | 0,02 | 0,04 | 0,09 | 0,06 |
| 9 | 3 | F | Nose | 0,2 | 0,04 | 0,04 | 0,07 | 0,06 | 0,02 | 0,18 | 0,1 |
| 10 | 35 | F | Eye | 0,08 | 0,14 | 0,03 | 0,04 | 0,04 | 0,05 | 0,11 | 0,11 |
| 11 | 22 | M | Ear | 0,02 | 0,02 | 0,03 | 0,01 | 0,09 | 0,08 | 0,1 | 0,12 |
| 12 | 38 | F | Ear | 0,14 | 0,1 | 0 | 0 | 0,00 | 0,03 | 0,01 | 0,01 |
| 13 | 27 | M | Lip | 0,05 | 0,06 | 0,05 | 0,01 | 0,02 | 0,02 | 0,09 | 0,05 |

**Additional File 1: Table S1**. M1 Score for 4 HR in EC between MVP and mature vessels area

**Additional File 1: Table S2**. M1 Score for 4 HR in SMC between MVP and mature vessels area

| **No** | **Age** | **Sex** | **Topography** | **Hormone Receptor** | | | | | | | |
| --- | --- | --- | --- | --- | --- | --- | --- | --- | --- | --- | --- |
|  |  |  |  | **ER** | | **PGR** | | **GHR** | | **FSHR** | |
|  |  |  |  | **MVP** | **Mature** | **MVP** | **Mature** | **MVP** | **Mature** | **MVP** | **Mature** |
| 1 | 2 | F | Arm | 0,51 | 0,45 | 0,34 | 0,33 | 0,45 | 0,64 | 0,31 | 0,55 |
| 2 | 18 | F | Nose | 0,21 | 0,33 | 0,26 | 0,47 | 0,24 | 0,33 | 0,42 | 0,34 |
| 3 | 16 | F | Foot | 0,18 | 0,35 | 0,48 | 0,45 | 0,61 | 0,38 | 0,24 | 0,39 |
| 4 | 48 | F | Face | 0,31 | 0,17 | 0,16 | 0,42 | 0,16 | 0,23 | 0,85 | 0,68 |
| 5 | 18 | M | Skin | 0,37 | 0,35 | 0,36 | 0,33 | 0,43 | 0,44 | 0,54 | 0,53 |
| 6 | 42 | F | Lip | 0,13 | 0,2 | 0,19 | 0,3 | 0,39 | 0,44 | 0,33 | 0,43 |
| 7 | 30 | M | Leg | 0,17 | 0,2 | 0,28 | 0,41 | 0,44 | 0,37 | 0,1 | 0,16 |
| 8 | 27 | F | Lip | 0,2 | 0,19 | 0,28 | 0,42 | 0,26 | 0,61 | 0,1 | 0,16 |
| 9 | 3 | F | Nose | 0,16 | 0,14 | 0,39 | 0,52 | 0,29 | 0,34 | 0,75 | 0,71 |
| 10 | 35 | F | Eye | 0,19 | 0,24 | 0,22 | 0,32 | 0,27 | 0,53 | 0,11 | 0,31 |
| 11 | 22 | M | Ear | 0,46 | 0,57 | 0,22 | 0,21 | 0,71 | 0,58 | 0,19 | 0,5 |
| 12 | 38 | F | Ear | 0,29 | 0,19 | 0,07 | 0,16 | 0,28 | 0,54 | 0,27 | 0,42 |
| 13 | 27 | M | Lip | 0,31 | 0,31 | 0,27 | 0,14 | 0,3 | 0,35 | 0,1 | 0,5 |

**Additional File 1: Table S3**. M2 Score for 4 HR in EC between MVP and mature vessels area

| **No** | **Age** | **Sex** | **Topography** | **Hormone Receptor** | | | | | | | |
| --- | --- | --- | --- | --- | --- | --- | --- | --- | --- | --- | --- |
|  |  |  |  | **ER** | | **PGR** | | **GHR** | | **FSHR** | |
|  |  |  |  | **MVP** | **Mature** | **MVP** | **Mature** | **MVP** | **Mature** | **MVP** | **Mature** |
| 1 | 2 | F | Arm | 0,33 | 0,34 | 0,12 | 0,08 | 0,15 | 0,2 | 0,21 | 0,33 |
| 2 | 18 | F | Nose | 0,06 | 0,26 | 0,14 | 0,16 | 0,2 | 0,29 | 0,47 | 0,3 |
| 3 | 16 | F | Foot | 0,54 | 0,33 | 0,08 | 0,11 | 0,21 | 0,11 | 0,13 | 0,09 |
| 4 | 48 | F | Face | 0,49 | 0,35 | 0,2 | 0,14 | 0,27 | 0,11 | 0,39 | 0,19 |
| 5 | 18 | M | Skin | 0,41 | 0,31 | 0,15 | 0,11 | 0,2 | 0,18 | 0,48 | 0,32 |
| 6 | 42 | F | Lip | 0,43 | 0,3 | 0,08 | 0,09 | 0,06 | 0,07 | 0,18 | 0,19 |
| 7 | 30 | M | Leg | 0,16 | 0,3 | 0,1 | 0,09 | 0,54 | 0,41 | 0,08 | 0,22 |
| 8 | 27 | F | Lip | 0,17 | 0,31 | 0,18 | 0,19 | 0,16 | 0,12 | 0,4 | 0,35 |
| 9 | 3 | F | Nose | 0,42 | 0,18 | 0,1 | 0,23 | 0,02 | 0,04 | 0,45 | 0,38 |
| 10 | 35 | F | Eye | 0,5 | 0,38 | 0,04 | 0,04 | 0,14 | 0,16 | 0,3 | 0,38 |
| 11 | 22 | M | Ear | 0,23 | 0,37 | 0,05 | 0,07 | 0,53 | 0,4 | 0,33 | 0,35 |
| 12 | 38 | F | Ear | 0,26 | 0,41 | 0,03 | 0,03 | 0,11 | 0,37 | 0,16 | 0,11 |
| 13 | 27 | M | Lip | 0,32 | 0,25 | 0,07 | 0,04 | 0,1 | 0,08 | 0,23 | 0,23 |

**Additional File 1: Table S4**. M2 Score for 4 HR in SMC between MVP and mature vessels area

| **No** | **Age** | **Sex** | **Topography** | **Hormone Receptor** | | | | | | | |
| --- | --- | --- | --- | --- | --- | --- | --- | --- | --- | --- | --- |
|  |  |  |  | **ER** | | **PGR** | | **GHR** | | **FSHR** | |
|  |  |  |  | **MVP** | **Mature** | **MVP** | **Mature** | **MVP** | **Mature** | **MVP** | **Mature** |
| 1 | 2 | F | Arm | 0,35 | 0,39 | 0,16 | 0,16 | 0,18 | 0,31 | 0,25 | 0,49 |
| 2 | 18 | F | Nose | 0,36 | 0,46 | 0,14 | 0,21 | 0,18 | 0,31 | 0,38 | 0,27 |
| 3 | 16 | F | Foot | 0,5 | 0,35 | 0,06 | 0,1 | 0,17 | 0,13 | 0,15 | 0,16 |
| 4 | 48 | F | Face | 0,51 | 0,41 | 0,17 | 0,32 | 0,18 | 0,09 | 0,32 | 0,2 |
| 5 | 18 | M | Skin | 0,44 | 0,36 | 0,14 | 0,1 | 0,16 | 0,18 | 0,35 | 0,27 |
| 6 | 42 | F | Lip | 0,44 | 0,28 | 0,1 | 0,12 | 0,07 | 0,15 | 0,21 | 0,22 |
| 7 | 30 | M | Leg | 0,15 | 0,33 | 0,1 | 0,09 | 0,54 | 0,42 | 0,11 | 0,28 |
| 8 | 27 | F | Lip | 0,22 | 0,41 | 0,2 | 0,2 | 0,14 | 0,15 | 0,37 | 0,33 |
| 9 | 3 | F | Nose | 0,45 | 0,24 | 0,1 | 0,22 | 0,06 | 0,12 | 0,35 | 0,32 |
| 10 | 35 | F | Eye | 0,46 | 0,44 | 0,03 | 0,05 | 0,12 | 0,16 | 0,24 | 0,34 |
| 11 | 22 | M | Ear | 0,26 | 0,36 | 0,04 | 0,07 | 0,53 | 0,4 | 0,31 | 0,33 |
| 12 | 38 | F | Ear | 0,47 | 0,58 | 0,04 | 0,04 | 0,13 | 0,49 | 0,21 | 0,18 |
| 13 | 27 | M | Lip | 0,34 | 0,26 | 0,06 | 0,07 | 0,12 | 0,08 | 0,19 | 0,21 |
